# Maternal exercise attenuates the lower skeletal muscle glucose uptake and insulin secretion caused by paternal obesity in female adult rat offspring

**DOI:** 10.1113/JP279582

**Published:** 2020-07-09

**Authors:** Filippe Falcão‐Tebas, Evelyn C. Marin, Jujiao Kuang, David J. Bishop, Glenn K. McConell

**Affiliations:** ^1^ Institute for Health and Sport (IHES) Victoria University Melbourne Australia; ^2^ The Ritchie Centre, Hudson Institute of Medical Research, and Department of Obstetrics and Gynaecology Monash University Melbourne Australia; ^3^ Department of Medicine, Austin Health The University of Melbourne Melbourne Australia; ^4^ College of Health and Biomedicine Victoria University Melbourne Australia

**Keywords:** developmental origins of health and disease (DOHaD), gestation, high‐fat diet, mitochondria, pancreas, physical activity, pregnancy, signalling, skeletal muscle

## Abstract

**Key points:**

- Paternal obesity negatively influences metabolic outcomes in adult rat offspring.
- Maternal voluntary physical activity has previously been reported to improve glucose metabolism in adult rat offspring sired by healthy fathers.
- Here, we investigated whether a structured programme of maternal exercise training before and during gestation can attenuate the negative impacts that paternal obesity has on insulin sensitivity and secretion in female adult offspring.
- Exercise before and during pregnancy normalised the lower insulin sensitivity in skeletal muscle and the lower insulin secretion observed in female offspring sired by obese fathers.
- This paper presents a feasible, low‐cost and translatable intervention strategy that can be applied perinatally to support multifactor interventions to break the cycle of metabolic dysfunction caused by paternal obesity.

**Abstract:**

We investigated whether maternal exercise before and during gestation could attenuate the negative metabolic effects of paternal high‐fat diet‐induced obesity in female adult rat offspring. Fathers consumed a normal chow or high‐fat diet before mating. Mothers exercised on a treadmill before and during gestation or remained sedentary. In adulthood, female offspring were assessed using intraperitoneal insulin and glucose tolerance tests (IPITT and IPGTT, respectively), pancreatic morphology, *ex vivo* skeletal muscle insulin‐stimulated glucose uptake and mitochondrial respiratory function. Paternal obesity impaired whole‐body and skeletal muscle insulin sensitivity and insulin secretion in adult offspring. Maternal exercise attenuated the lower insulin‐stimulated glucose uptake in offspring sired by obese fathers but distal insulin signalling components (p‐AKT Thr308 and Ser473, p‐TBC1D4 Thr642 and GLUT4) remained unchanged (*P* > 0.05). Maternal exercise increased citrate synthase activity only in offspring sired by obese fathers. Maternal exercise also reversed the lower insulin secretion *in vivo* observed in offspring of obese fathers, probably due to an attenuation of the decrease in pancreatic beta cell mass. In summary, maternal exercise before and during pregnancy in rats attenuated skeletal muscle insulin resistance and attenuated the decrease in pancreatic beta cell mass and insulin secretion observed in the female offspring of obese fathers.

## Key points

- Paternal obesity negatively influences metabolic outcomes in adult rat offspring.
- Maternal voluntary physical activity has previously been reported to improve glucose metabolism in adult rat offspring sired by healthy fathers.
- Here, we investigated whether a structured programme of maternal exercise training before and during gestation can attenuate the negative impacts that paternal obesity has on insulin sensitivity and secretion in female adult offspring.
- Exercise before and during pregnancy normalised the lower insulin sensitivity in skeletal muscle and the lower insulin secretion observed in female offspring sired by obese fathers.
- This paper presents a feasible, low‐cost and translatable intervention strategy that can be applied perinatally to support multifactor interventions to break the cycle of metabolic dysfunction caused by paternal obesity.

## Introduction

The developmental origins of health and disease (DOHaD) paradigm focuses on investigating how environmental cues acting during early development affect health status in adulthood (McMullen & Mostyn, 2009; Hanson & Gluckman, 2014; Street *et al*. 2015). Much of the research is focused on maternal effects (reviewed by (McMullen & Mostyn, 2009; Warner & Ozanne, 2010; Gatford *et al*. 2014), with the contribution of fathers to metabolic diseases such as type 2 diabetes mellitus only recently receiving attention in the DOHaD field.

Paternal obesity before conception can negatively influence the phenotype and metabolism of the offspring (Soubry, 2015). In fact, a father's body fat is predictive of long‐term alterations in total and percentage body fat in their prepubertal daughters (Figueroa‐Colon *et al*. 2000). In rats, Ng *et al*. (2010) have demonstrated that a paternal high‐fat diet for 10 weeks before conception does not affect body mass or fat mass in female offspring (Ng *et al*. 2010). In this study, however, during an intraperitoneal glucose tolerance test (IPGTT) the offspring demonstrated lower insulin area under the curve and higher glucose area under the curve compared to offspring sired by control‐diet fathers at 12 weeks of age. These findings show that adolescent offspring (12 weeks of age) developed glucose intolerance in addition to altered pancreatic islet morphology, including reduced total islet area and higher percentage of small islets. The study also reported altered gene expression in several pathways in the pancreatic islets of female offspring, including MAPK and Jak–Stat signalling, cell cycle and apoptosis (Ng *et al*. 2010). Similar negative effects were reported in mice sired from obese fathers (high‐fat diet with 40% energy as fat) in terms of glucose metabolism in adulthood (Fullston *et al*. 2013). We have demonstrated that whole‐body insulin sensitivity, insulin‐stimulated glucose uptake and mitochondrial respiration in skeletal muscle as well as insulin secretion were negatively affected in adult female offspring sired by obese fathers (two high‐fat diets with 40.7% and 43.0% energy as fat) (Falcao‐Tebas *et al*. 2019).

Maternal phenotype plays an essential role in an offspring's health, as the fetus experiences direct interaction with the mother through the placenta (Power & Schulkin, 2013). This relationship opens up possible ways to intervene during pregnancy, such as having a balanced diet as well as promoting physical activity. Maternal exercise studies in healthy humans have, however, produced conflicting results, and report reduced (Clapp & Capeless, 1990; Hopkins *et al*. 2010), no effect (Marquez‐Sterling *et al*. 2000) or increased birth weight (Clapp *et al*. 2000, 2002). This is probably due to different exercise intensities, volume, fitness levels of the mothers and the time point in the pregnancy when the exercise is performed (Clapp *et al*. 2002; Clapp, 2003). Using rodent models of voluntary running wheel exercise, other research groups have demonstrated that maternal exercise can improve offspring glucose tolerance later in life compared to the offspring of control/sedentary mothers (Carter *et al*. 2012, 2013; Laker *et al*. 2014; Stanford *et al*. 2015, 2017). When performing a structured training programme on a treadmill in rats, we have shown that exercise before and during pregnancy improved glucose homeostasis in the short and long term in offspring of undernourished mothers (Fidalgo *et al*. 2013; Leandro *et al*. 2012; Falcao‐Tebas *et al*. 2012 *b*). Other groups have shown that maternal exercise may also improve other health outcomes in adult offspring, for example by protecting against maternal obesity‐induced metabolic dysfunction in skeletal muscle (Laker *et al*. 2014).

There are, however, gaps in the literature related to the combined or isolated effects of maternal exercise and/or paternal obesity on offspring later in life. For example, there is limited information regarding the influence of maternal exercise or paternal obesity on glucose regulation and mitochondrial function in the skeletal muscle of adult offspring. In terms of insulin secretion, one publication reported that exercise training (treadmill) before and during gestation does not alter pancreatic insulin content, but augments insulin secretory capacity in young rats (3 weeks of age) while reducing it with ageing (∼28 weeks of age) (Quiclet *et al*. 2016). Whether exercise can be used to prevent the negative effects of paternal obesity on the offspring is yet to be investigated.

Therefore, here we investigated whether maternal exercise before and during gestation could attenuate the negative impacts on insulin sensitivity and secretion in adult offspring caused by paternal obesity and the underlying mechanisms. We hypothesised that maternal exercise attenuates the adverse metabolic effects of paternal obesity in adult offspring by improving glucose sensitivity and mitochondrial respiration in skeletal muscle as well as normalising beta cell mass.

## Research design and methods

### Ethical approval

All procedures were performed according to the Australian Code for the Care and Use of Animals for Scientific Purposes (2013), after approval by Victoria University Animal Ethics Committee (#13/008). Sprague Dawley (ArcCrl:CD(SD)IGS) breeders were obtained from the Animal Resources Centre (Murdoch, Western Australia, Australia) and all animals were kept at the Victoria University Footscray Park Campus Animal Facility on a 12:12 h light–dark cycle with free access to food and water. The authors understand the ethical principles under which *The Journal of Physiology* operates and confirm that this work meets the standards of the journal's animal ethics checklist.

### Animals

**Figure 1:**
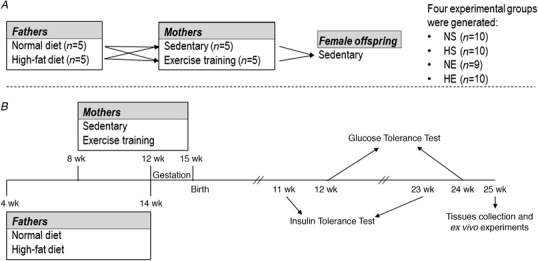
Experimental groups and timelines *A*, experimental groups based on paternal diet and maternal exercise interventions. Normal‐diet and high‐fat diet groups were used in our previous paper (Falcao‐Tebas *et al*. 2019). Male breeders were mated with only one female (1:1). *B*, experimental design with timelines for main experiments. NS, offspring sired by normal‐diet fathers and sedentary mothers. HS, offspring sired by obese fathers and sedentary mothers. NE, offspring sired by normal‐diet fathers and exercised mothers. HE, offspring sired by obese fathers and exercised mothers.

In this study, we used a model of high‐fat diet‐induced paternal obesity. Male rats were fed a control chow diet (*n* = 5; Rat and Mouse Cubes, 12.0% energy as fat; Specialty Feeds, Western Australia) or high‐fat diets (*n* = 5; SF01‐025 and SF03‐020, with 40.7% and 43.0% energy as fat; Specialty Feeds) from 4 to 14 weeks of age (Fig. 1). Two types of high‐fat chow were used to match the dietary protocol used by Ng *et al*. (2010). This paper forms part of a larger study and, as such, two groups [offspring sired by control diet fathers and sedentary mothers (NS); offspring sired by obese fathers and sedentary mothers (HS)] were presented in our paper examining the effects of exercise early in life of offspring sired by obese fathers (Falcao‐Tebas *et al*. 2019). Importantly, all experimental groups in this project were created and studied at the same time. Each male impregnated one female (1:1). Four dams did not impregnate within a suitable time frame and were not included in the study. All animals were monitored and had their health assessed daily.

Female breeders were obtained at 6 weeks of age and maintained on a control chow diet. All female breeders were acclimatised to the treadmill with five sessions of exercise over 3 days, and randomly placed in sedentary (*n* = 5) or exercise training (*n* = 5) groups. Mating was performed when rats were 12 weeks old. From 08.00 to 17.00 h, females were moved into the male cages. During this time, only control chow diet was available. Females continued to exercise during the mating period until pregnancy was confirmed by vaginal smear. Once pregnant, dams were housed individually. The exercise group continued performing exercise training, with a progressive reduction in intensity and volume until day 19 of gestation (Table 1).

**Table 1:** Exercise training protocol before and during pregnancy

	Week no.	Speed (m/s)	Grade (°)	Percentage of ${\dot{V}}_{O_{2}\max}$ ^*^	Duration of each stage (min)	Total session time (min)
Before gestation	1	4.98	0	∼60	5	50
		12.6	0		40	
		4.98	0		5	
	2	6.66	5.0	∼64	5	60
		15.18	5.0		50	
		6.66	5.0		5	
	3	8.28	10.0	∼70	5	60
		17.40	10.0		50	
		8.28	10.0		5	
	4	8.28	10.0	∼76	5	60
		19.26	10.0		50	
		8.28	10.0		5	
During gestation	1	8.28	5.0	∼65	5	50
		15.18	5.0		40	
		8.28	5.0		5	
	2	8.28	5.0	∼58	5	40
		12.00	5.0		30	
		8.28	5.0		5	
	3	4.98	0	∼50	5	30
		10.02	0		20	
		4.98	0		5	

* Estimated percentage of ${\dot{V}}_{O_{2}\max}$ based on previous studies (Bedford *et al*. 1979; Amorim *et al*. 2009).

This study only included litter sizes between nine and 15 pups. Birth weight refers to body mass on postnatal day 1. At postnatal day 1, litters were standardised to 12 pups, with six males and six females when possible. After weaning (postnatal day 21), only two female pups were included in this study from each litter in an attempt to replicate and expand previous findings (Ng *et al*. 2010). There were no interventions in the offspring. We have defined adolescent offspring as 11–12 weeks of age and adult offspring as 23–24 weeks of age (Falcao‐Tebas *et al*. 2019). All *in vivo* and *in vitro* procedures were conducted between 07.00 and 13.00 h. Offspring were killed by cardiac puncture at 25 weeks of age. All analyses were conducted under blinded conditions using coded samples.

### Insulin tolerance and glucose tolerance tests

Intraperitoneal insulin tolerance tests (IPITT; 1 U/kg body mass) and intraperitoneal glucose tolerance tests (IPGTT; 1.0 g/kg body mass) were performed in adolescent and adult rats (Fig. 1) as described previously (Ng *et al*. 2010; Laker *et al*. 2011; Falcao‐Tebas *et al*. 2019). Tail vein blood glucose was measured via a glucometer (Accu‐Chek Performa Nano, Roche Diagnostics, Mannheim, Germany). Insulin was analysed by radioimmunoassay (SRI‐13K RI‐13K, Linco Research, St Charles, MO, USA).

The insulinogenic index, a surrogate marker of insulin secretion during the first‐phase insulin response to a glucose challenge, was derived from data obtained from the IPGTT that was conducted at 24 weeks of age. It was calculated by dividing the area under the curve (AUC) for plasma insulin levels (0–30 min) by the AUC for plasma glucose levels (0–30 min) (Ng *et al*. 2010).

### Insulin‐stimulated glucose uptake in skeletal muscle

At 25 weeks of age, the offspring were anaesthetised (60 mg kg^−1^ i.p.; pentobarbitone; Virbac, Milperra, NSW, Australia) and checked every 10 min by performing a tail pinch and observing no change in the respiratory rate. Epitrochlearis and soleus muscles were dissected and longitudinally split in half (Sharma *et al*. 2015). We used the radioactive glucose analogue [1,2‐^3^H]2‐deoxy‐glucose ([^3^H]2‐DG), and radioactive mannitol, d‐[^14^C] mannitol ([^14^C]mannitol), to measure glucose transport *in vitro* into skeletal muscle (Hansen *et al*. 1994). Muscles were incubated in chambers filled with Krebs Henseleit solution (in mm): 118.5 NaCl, 24.7 NaHCO_3_, 4.74 KCl, 1.18 MgSO_4_, 1.18 KH_2_PO_4_, 147.02 CaCl_2_, 32 mannitol, 7.5% bovine serum albumin (BSA) and MilliQ H_2_O, with pH 7.4, maintained at 30°C and continuously oxygenated with 95% O_2_ and 5% CO_2_ (Falcao‐Tebas *et al*. 2019). During the first 20 min, the muscles were incubated with the Krebs Henseleit solution, in addition to 8 mm glucose. Then, muscles were transferred to another Krebs Henseleit solution for 30 min, with 4 mm pyruvate and no glucose. This second buffer also contained either 0 or 1.2 nm insulin. Next, muscles were transferred to a buffer comprising the Krebs Henseleit solution with the addition of 8 mm 2‐deoxyglucose (0.75 µCi/ml 2‐[1,2‐^3^H] deoxy‐d‐glucose), 2 mm mannitol (0.225 µCi/ml [1‐^14^C]mannitol), and 0 or 1.2 nm insulin for 10 min. At the end of the incubation protocol, the muscles were quickly washed in ice‐cold Krebs Henseleit solution, rapidly dried on filter paper three times, and frozen in liquid nitrogen (Falcao‐Tebas *et al*. 2019).

Approximately 30 mg of epitrochlearis and 35 mg of soleus muscles were digested with 300 µl of 1 m NaOH at 95°C for 10 min, neutralised with 300 µl of 1 m HCl, vortexed and then centrifuged for 5 min at 13,000 *g* at room temperature. The homogenate was transferred into the glass scintillation vial containing 4 ml of biodegradable scintillation cocktail (Ultima Gold, PerkinElmer, Boston, MA, USA). After 1 h at room temperature, the samples were read on a β‐scintillation counter (Liquid Scintillation Analyser, Tri‐Carb 2810TR, PerkinElmer) with channels pre‐set for simultaneous measurement of [^3^H]2‐DG and [^14^C]mannitol with each sample read for 10 min (Stephens *et al*. 2004).

To calculate skeletal muscle glucose uptake, [^14^C]mannitol counts were subtracted from the [^3^H]2‐DG counts in muscle homogenates to provide an index for intracellular phosphorylation of [^3^H]2‐DG accumulation and an estimate of skeletal muscle glucose uptake (Narahara & Ozand, 1963; Hansen *et al*. 1994). We used liquid scintillation counting of skeletal muscle homogenates to determine the extracellular space by quantifying [^14^C]mannitol counts/min. The difference between the total [^3^H]2‐DG in muscle and the [^3^H]2‐DG in the extracellular space was considered the intracellular [^3^H]2‐DG of the muscle. The following formula was then used:

$$\begin{array}{ccc} & & \mathrm{Glucose}\;\mathrm{uptake}\\ & & \;=\left(\left[3H\right]2\mathrm{DG}\;\mathrm{intracellular}+\mathrm{extracellular}\;\left(\mu \mathrm{mol}\right)\right)\\ & & \;-\left(\left(\left[3H\right]2\mathrm{DG}\;\mathrm{intracellular}\left(\mu \mathrm{mol}\right)\right)\right)/\\ & & \;\;\left(\mathrm{Muscle}\;\mathrm{mass}\left(g\right)*1h\right) \end{array}$$

### Skeletal muscle mitochondrial respiration and reactive oxygen species production

Approximately 2 mg of plantaris muscle was placed in cold Biopsy Preservation Solution (BIOPS) as described previously (Pesta & Gnaiger, 2012; Falcao‐Tebas *et al*. 2019). Muscle fibres were mechanically separated under a microscope prior to chemical permeabilisation of the plasma membrane (30 min incubation, 50 µg/ml saponin). Mitochondrial respiration was performed on muscle fibre bundles by high‐resolution respirometry with an Oxygraph‐2k (O2k, OROBOROS Instruments, Innsbruck, Austria) (Pesta & Gnaiger, 2012) combined with the Fluorescence‐Sensor Green of the O2k‐Fluo LED2‐Module for H_2_O_2_ measurement. The substrate–uncoupler–inhibitor titration protocol was performed as described elsewhere (Falcao‐Tebas *et al*. 2019). We measured leak respiration (*L*) through complex I (CI) (CI_L_), maximum oxidative phosphorylation (oxphos) capacity (*P*) through CI (CI_P_), *P* through CI+II combined (CI+II_P_), electron transport system (ETS) capacity (*E*) through CI+II (CI+II_E_), *E* through CII (CII_E_) and finally residual oxygen consumption (ROX) and emission of H_2_O_2_ (Pesta & Gnaiger, 2012).

The citrate synthase (CS) assay was adapted to be performed on a 96‐well plate (Srere, 1969; McConell *et al*. 2015), by examining the increase of 5,5‐dithiobis‐2‐nitrobenzoate (DTNB) at a wavelength of 412 nm. We have considered CS activity as a marker of mitochondrial volume in skeletal muscle (Larsen *et al*. 2012). Mitochondrial respiration was expressed as mass‐specific respiration (normalised by muscle mass) or mitochondrial‐specific respiration (obtained by normalising mass‐specific mitochondrial respiration by CS activity).

### Western blots

Epitrochlearis muscle with and without insulin, and plantaris muscle, were homogenised in ice‐cold buffer, and lysates were prepared as described previously (Betteridge *et al*. 2016; Falcao‐Tebas *et al*. 2019). Western blots were carried out using hand‐cast TGX Stain‐Free gels (Bio‐Rad, Hercules, CA, USA) loaded with 4 µg of skeletal muscle lysate and transferred to polyvinylidene difluoride membranes (Bio‐Rad). After protein transfer, membranes were imaged to quantify total protein using Imagelab 4.1 (Bio‐Rad) (Murphy, 2011). After overnight incubation with primary antibodies, membranes were exposed to SuperSignal West Femto Maximum Sensitivity Substrate (Thermo Fisher Scientific, Waltham, MA, USA) and the bands were analysed with ImageLab software (Version 5.2.1, Bio‐Rad).

Antibodies used were anti‐GLUT4 (Abcam Cat. No. ab37445, RRID:AB_732612; Cambridge, MA, USA), anti‐GLUT1 (Abcam Cat. No. ab652, RRID:AB_305540), AKT2 (Cell Signaling Technology Cat. No. 5239, RRID:AB_10544406; Danvers, MA, USA), anti‐TBC1D4 (Cell Signaling Technology Cat. No. 2670, RRID:AB_2199375), anti‐Tfam (Abcam Cat. No. ab131607, RRID:AB_11154693), anti‐PGC1α (Millipore Cat. No. ST1202, RRID:AB_2237237; Billerica, MA, USA), and anti‐PHF20 (Cell Signaling Technology Cat. No. 3934, RRID:AB_2165078). The primary phosphorylated (p)‐specific antibodies were anti‐p‐AS160 Thr642 (Cell Signaling Technology Cat. No. 4288, RRID:AB_10545274), anti‐p‐AKT Thr308 (Cell Signaling Technology Cat. No. 9275, RRID:AB_329828) and anti‐p‐AKT Ser473 (Cell Signaling Technology Cat. No. 9271, RRID:AB_329825).

### Pancreas morphology

After dissection of skeletal muscles, rats were killed by cardiac puncture, and pancreases were removed, weighed and stored in 10% neutral buffered formalin, and then transferred to 70% ethanol until processed. Five sections per pancreas were immunostained to identify and localise insulin‐positive beta cells (*n* = 8–10 per group) (Falcao‐Tebas *et al*. 2019). Fixed tissue was sent to Anatomical Pathology, Department of Medicine, University of Melbourne (Parkville, Victoria, Australia) to be paraffin embedded, sectioned at 100 µm and stained for insulin using a guinea pig polyclonal anti‐porcine insulin antibody (DAKO Corporation, Denmark) diluted 1:100 and counterstained with haematoxylin. High‐resolution images of microscopic sections were obtained through the Austin Health, Victorian Cancer Biobank Slide Scanning service (Heidelberg, Victoria, Australia). Following standard protocols, whole‐slide sections were line scanned using an Aperio ScanScope XT (Aperio Technologies, Vista, CA, USA) at 40× magnification at a resolution of 0.5 µm/pixel. Digital images were analysed using the Aperio image software (ImageScope version 12.2.2).

Measurements were performed as described previously (Laker *et al*. 2011; Falcao‐Tebas *et al*. 2019). Briefly, pancreatic islet number was expressed relative to total cross‐sectional area (mm^2^) with islet size arbitrarily classified as small (<5000 µm^2^), medium (5000–10,000 µm^2^) and large (>10,000 µm^2^). Pancreatic sections were analysed in whole, and relative islet and beta‐cell volume density (*Vd*) were quantified by point‐counting morphometry (*Vd* = number of intercepts on islet or insulin‐positive cells as a proportion of intercepts on pancreas). The number of islets was obtained by dividing the number of islets by the total pancreas area (mm^2^). Beta‐cell mass was determined by multiplying *Vd* by pancreas weight (mg).

### Statistical analyses

Data are reported as mean ± SD. Normality was tested using the Shapiro–Wilk test and data were log transformed and re‐analysed if applicable. Statistical analyses were performed using SPSS (IBM SPSS Statistics for Windows, Version 24.0, IBM Corp., Armonk, NY, USA) and GraphPad Prism (GraphPad Prism version 7.00 for Windows, GraphPad Software, La Jolla, CA, USA) using Student's *t* tests, a two‐ or three‐way ANOVA with repeated measures, as appropriate. For the ANOVA, ‘Paternal diet’ and ‘Maternal exercise’ were used as main factors, as well as time‐points (e.g. during the IPGTT and IPITT) or treatment (e.g. insulin incubation during 2DG uptake). If an interaction was found in a three‐way ANOVA, a two‐way ANOVA was applied to each time‐point/treatment. For two‐way ANOVA, if an interaction was found, a *post hoc* analysis using the least significant difference (LSD) test was used. The α‐level of statistical significance was set a priori at *P* < 0.05.

## Results

As this study was part of a larger project, data from two groups (NS and HS) were presented in our paper examining the effects of paternal obesity on offspring exercise (Falcao‐Tebas *et al*. 2019). Here, we will focus on the effects of maternal exercise in the female offspring. Maternal exercise did not alter food intake or body mass before and during gestation or lactation (data not shown).

### Birth weight, weaning and growth

Paternal obesity did not change the number of male and female pups born, birth weight or body mass until postnatal day 21 (*P* > 0.05) (Table 2). At postnatal day 21, paternal obesity did not affect liver, spleen, heart, kidneys, pancreas or extensor digitorum longus muscle mass (*P* > 0.05), while soleus muscle mass was reduced. Maternal exercise also had no effects on the number or sex of pups born, or their birth weight (*P* > 0.05). A small reduction (*P* < 0.05) in weaning body mass was observed in offspring sired by exercised mothers, independent of paternal obesity (Table 2).

**Table 2:** Characteristics at birth (PND1) and weaning (PND21) of offspring sired by control or obese fathers and sedentary or exercised mothers

Parameter	NS (*n* = 5 litters)	HS (*n* = 5 litters)	NE (*n* = 5 litters)	HE (*n* = 5 litters)
Number of pups born per litter	13.0 ± 3.3	14.8 ± 1.6	12.60 ± 1.5	14.00 ± 1.6
Number of females per litter	5.50 ± 1.1	7.60 ± 2.3	7.20 ± 1.3	8.25 ± 2.9
Number of males per litter	7.50 ± 2.7	7.20 ± 1.6	5.40 ± 1.5	5.75 ± 1.9
PND1 body mass females (g)	7.23 ± 0.2	7.02 ± 0.4	8.09 ± 1.2	6.93 ± 1.2
PND1 body mass of males (g)	7.69 ± 0.7	7.35 ± 0.4	8.55 ± 1.3	6.56 ± 1.0
PND1 blood glucose (mmol/l) ^*^	4.67 ± 1.1	5.20 ± 0.6	5.25 ± 1.7	4.90 ± 1.3
**Measurements at PND21 (female offspring)**				
Body mass (g)	53.94 ± 1.7	52.56 ± 2.2	51.53 ± 2.1^§^	47.36 ± 1.9^§^
Body length (cm)	12.81 ± 0.2	12.80 ± 0.3	12.95 ± 0.2	12.53 ± 0.4
Liver (g)	2.29 ± 0.1	2.17 ± 0.2	2.25 ± 0.3	2.15 ± 0.5
Spleen (g)	0.24 ± 0.0	0.22 ± 0.1	0.21 ± 0.0	0.20 ± 0.1
Heart (g)	0.28 ± 0.0	0.25 ± 0.0	0.31 ± 0.1	0.25 ± 0.1
EDL (g)	0.03 ± 0.0	0.02 ± 0.0	0.03 ± 0.0	0.03 ± 0.0
Soleus (g)	0.03 ± 0.0	0.02 ± 0.0^‡^	0.03 ± 0.0	0.03 ± 0.0^+^
Kidney (g)	0.31 ± 0.0	0.30 ± 0.0	0.30 ± 0.0	0.27 ± 0.0
Pancreas (g)	0.21 ± 0.0	0.28 ± 0.1	0.22 ± 0.0	0.20 ± 0.0
Blood glucose (mmol/l)^†^	8.87 ± 2.9	8.08 ± 4.1	9.03 ± 5.1	8.40 ± 4.6

Sample sizes were considered per litter, not number of individual pups. PND, postnatal day. Values are presented as mean ± SD (except ranges shown for pups born, number of males, number of females; a Mann–Whitney U test was used in these cases).

* No fasting period.

† Blood glucose collected after a 4 h fasting period. EDL, extensor digitorum longus. NS, offspring sired by control‐diet fathers and sedentary mothers; HS, offspring sired by obese fathers and sedentary mothers; NE, offspring sired by control diet fathers and exercised mothers; HE, offspring sired by obese fathers and exercised mothers. Values are presented as mean ± SD.

‡ Paternal obesity effect, *P* < 0.05.

§ Maternal exercise effect, *P* < 0.05. ^+^Paternal obesity *vs*. Maternal exercise interaction, *P* < 0.05, followed by a least significant difference test (HS *vs*. HE).

Body mass, and soleus and gastrocnemius skeletal muscle masses were lower in adult offspring sired by obese fathers. Maternal exercise decreased tibialis anterior mass and had no effect on gastrocnemius mass, but increased soleus, plantaris and liver masses in adult female offspring (Table 3).

**Table 3:** Effects of maternal exercise on adult (25 weeks of age, post‐mortem) female offspring sired by obese fathers

	NS	HS	NE	HE
Parameter	*n* = 10	*n* = 10	*n* = 9	*n* = 10
Body mass (g)	333.5 ± 44.3	299.1 ± 22.1^†^	317.2 ± 38.4	315.3 ± 30.0^+^
Length (cm)	24.3 ± 0.6	24.0 ± 0.3	24.6 ± 0.6	24.3 ± 0.3
Abdominal circumference (cm)	18.3 ± 1.3	18.2 ± 0.6	17.9 ± 0.9	17.7 ± 0.9
Soleus (mg)	151.4 ± 18.7	130.9 ± 19.9^†^	150.1 ± 10.5	149.6 ± 11.1^+^
EDL (mg)	160.6 ± 22.1	149.2 ± 15.5	159.7 ± 16.5	153.4 ± 5.4
Plantaris (mg)	337.0 ± 36.0	314.4 ± 24.0^†^	328.8 ± 38.7	335.0 ± 14.2^+^
Tibialis anterior (mg)	669.4 ± 84.1	638.2 ± 34.5	566.6 ± 59.7^‡^	632.4 ± 48.4
Gastrocnemius (g)	1.7 ± 0.3	1.6 ± 0.0	1.7 ± 0.3	1.6 ± 0.0
Liver (g)	8.5 ± 0.9	8.1 ± 0.6	8.7 ± 1.2	9.1 ± 1.3^+^
Pancreas (g)	1.4 ± 0.3	1.2 ± 0.3	1.4 ± 0.3	1.2 ± 0.0
Retroperitoneal fat (g)	6.2 ± 0.9	6.9 ± 1.9	6.4 ± 4.2	6.4 ± 4.7
Kidney (g)^*^	1.0 ± 0.0	0.9 ± 0.0^†^	0.9 ± 0.0	0.9 ± 0.0
Heart (g)	1.0 ± 0.0	0.9 ± 0.0	1.0 ± 0.0	1.0 ± 0.0
Fasting glucose (mmol/l)	5.66 ± 0.3	5.63 ± 0.9	5.90 ± 1.1	5.75 ± 0.4
Fasting insulin (ng/ml)	0.15 ± 0.0	0.13 ± 0.1	0.14 ± 0.1	0.11 ± 0.1

EDL, extensor digitorum longus.

* The left and right kidneys were combined as no statistical difference was found between them. NS, offspring sired by control diet fathers and sedentary mothers; HS, offspring sired by obese fathers and sedentary mothers; NE, offspring sired by control‐diet fathers and exercised mothers; HE, offspring sired by obese fathers and exercised mothers. Values are presented as mean ± SD.

† Paternal obesity effect, *P* < 0.05.

‡ Maternal exercise effect, *P* < 0.05. ^+^Paternal obesity *vs*. Maternal exercise interaction, *P* < 0.05, followed by LSD test (HS *vs*. HE).

### Whole‐body insulin sensitivity and secretion

**Figure 2:**
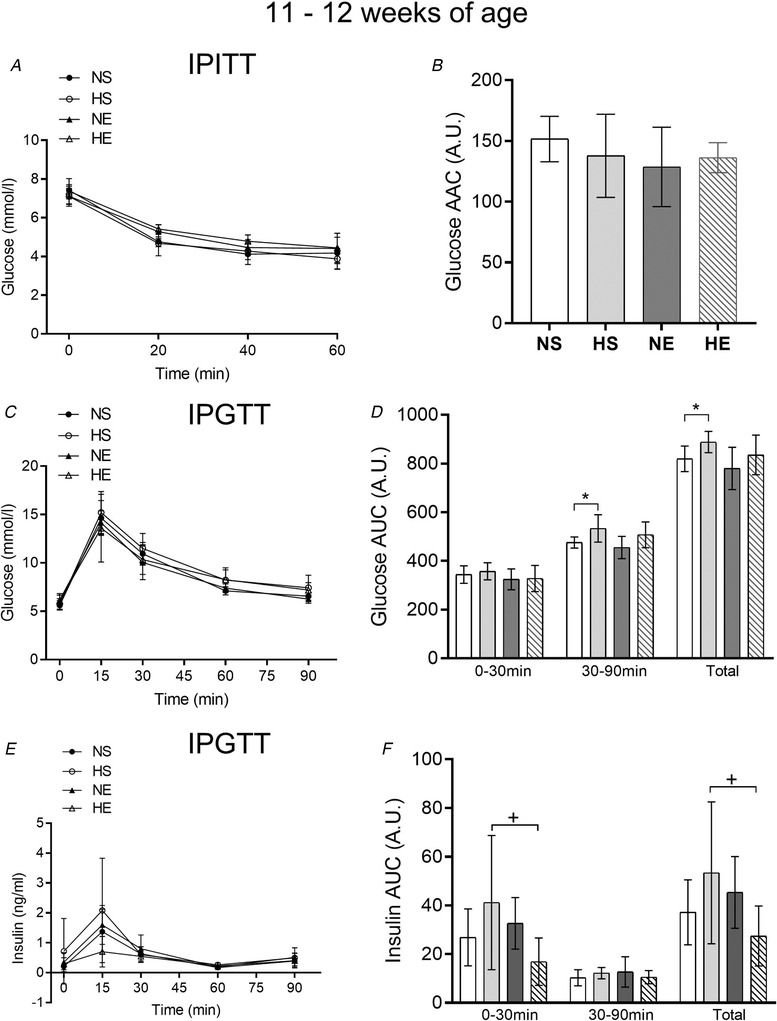
Insulin and glucose tolerance tests in adolescent offspring sired by control or obese fathers and sedentary or exercised mothers Glucose levels during an intraperitoneal insulin tolerance test (IPITT; *A*) and glucose (*C*) and insulin (*E*) levels during an intraperitoneal glucose tolerance test (IPGTT). Area above (AAC) and under (AUC) the curve for IPITT (*B*) and IPGTT (*D*, *F*), respectively. NS, offspring sired by control‐diet fathers and sedentary mothers (*n* = 10); HS, offspring sired by obese fathers and sedentary mothers (*n* = 10); NE, offspring sired by control diet fathers and exercised mothers (*n* = 9); HE, offspring sired by obese fathers and exercised mothers (*n* = 10). Values are presented as mean ± SD. ^*^Paternal obesity effect, *P* < 0.05. ^#^Maternal exercise effect, *P* < 0.05. ^+^Paternal obesity *vs*. Maternal exercise interaction, *P* < 0.05, followed by a least significant difference test (HS *vs*. HE).

Paternal obesity impaired whole‐body insulin sensitivity (estimated by IPITT), glucose tolerance and insulin secretion in adolescent offspring (11–12 weeks of age, Fig. 2). Maternal exercise had no effects on whole‐body insulin sensitivity, but improved glucose tolerance in the offspring of a normal‐weight father (Fig. 2*C* and *D*). Maternal exercise decreased the insulin AUC in adolescent female offspring sired by obese fathers (HE *vs*. HS group, Fig. 2*E* and *F*).

**Figure 3:**
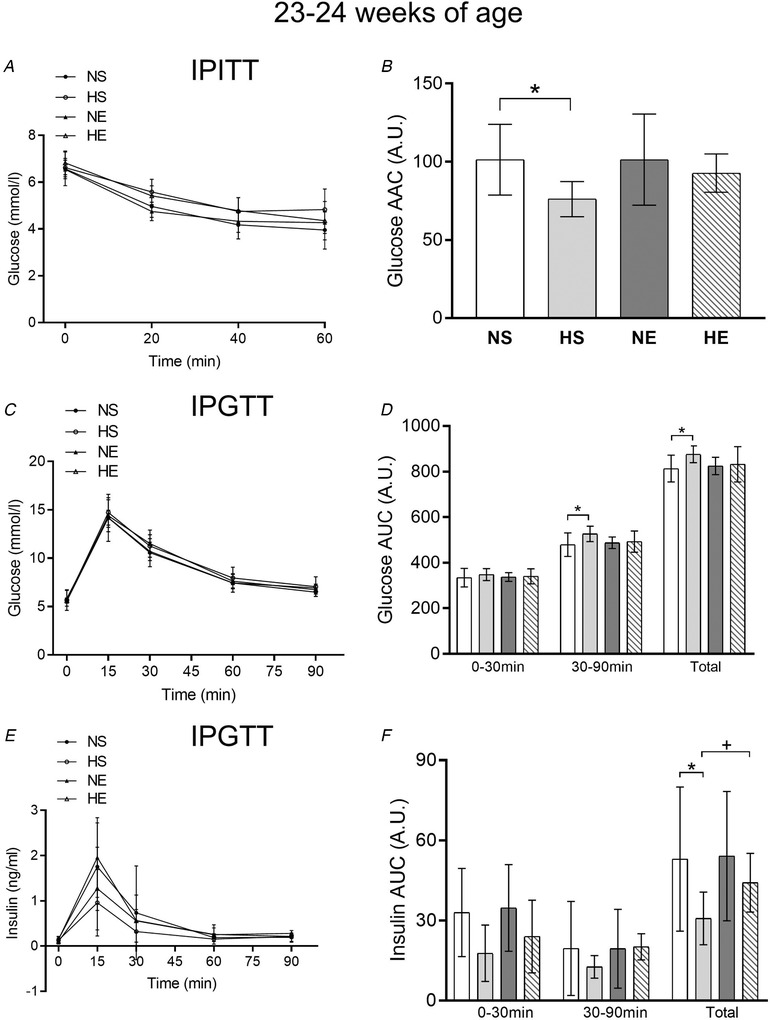
Insulin and glucose tolerance tests in adult offspring sired by control or obese fathers and sedentary or exercised mothers Glucose levels during an intraperitoneal insulin tolerance test (IPITT; *A*) and glucose (*C*) and insulin (*E*) levels during an intraperitoneal glucose tolerance test (IPGTT). Area above (AAC) and under (AUC) the curve for IPITT (*B*) and IPGTT (*D*, *F*), respectively. NS, offspring sired by control diet fathers and sedentary mothers (*n* = 10); HS, offspring sired by obese fathers and sedentary mothers (*n* = 10); NE, offspring sired by control diet fathers and exercised mothers (*n* = 9); HE, offspring sired by obese fathers and exercised mothers (*n* = 10). Values are presented as mean ± SD. ^*^Paternal obesity effect, *P* < 0.05. ^#^Maternal exercise effect, *P* < 0.05. ^+^Paternal obesity *vs*. Maternal exercise interaction, *P* < 0.05, followed by a least significant difference test (HS *vs*. HE).

Paternal obesity impaired whole‐body insulin sensitivity, glucose tolerance and insulin secretion in adult rats (23–25 weeks of age) (Fig. 3). Maternal exercise had no influence on whole‐body insulin sensitivity (IPITT; Fig. 3*B*) or glucose AUC during the glucose tolerance test in adult female offspring (Fig. 3*D*). Maternal exercise attenuated the lower insulin levels during the glucose tolerance test observed in offspring from obese fathers (insulin AUC) (Fig. 3*F*).

### Skeletal muscle glucose uptake and protein expressions

**Figure 4:**
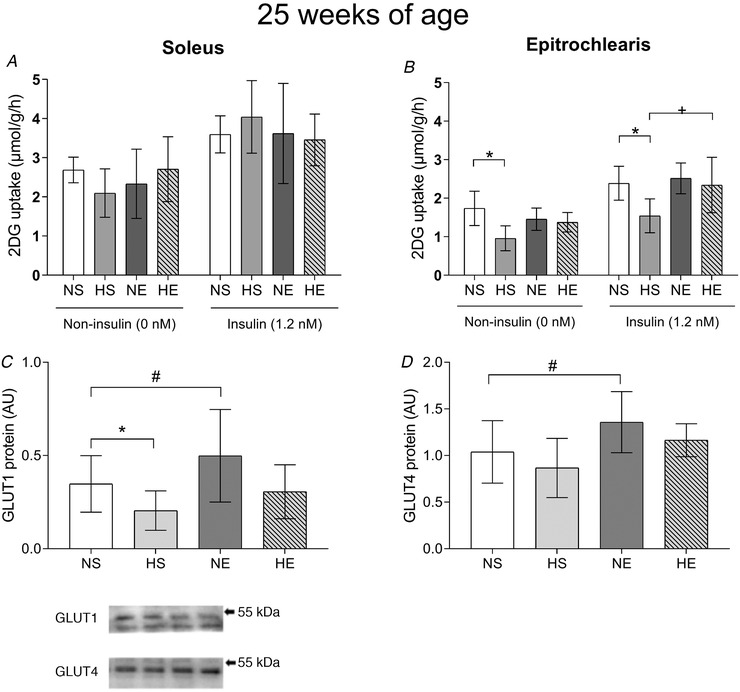
Insulin‐stimulated glucose uptake and protein expression of glucose transporters in epitrochlearis muscle of adult offspring sired by control or obese fathers and sedentary or exercised mothers Soleus (*A*) and epitrochlearis (*B*) muscle glucose uptake and protein expression of GLUT1 (*C*) and GLUT4 (*D*). Representative western blots show the quality and signal obtained with the respective antibodies; because they represent one animal, they do not necessarily represent an exact mean of their experimental group. NS, offspring sired by control diet fathers and sedentary mothers (*n* = 10); HS, offspring sired by obese fathers and sedentary mothers (*n* = 10); NE, offspring sired by control diet fathers and exercised mothers (*n* = 9); HE, offspring sired by obese fathers and exercised mothers (*n* = 10). Values are presented as mean ± SD. ^*^Paternal obesity effect, *P* < 0.05. ^#^Maternal exercise effect, *P* < 0.05. ^+^Paternal obesity *vs*. Maternal exercise interaction, *P* < 0.05, followed by a least significant difference test (HS *vs*. HE).

At 25 weeks of age, basal and insulin‐stimulated glucose uptake *ex vivo* in soleus muscle were not affected by any treatments (Fig. 4*A*). On the other hand, glucose uptake in epitrochlearis muscle of offspring sired by obese fathers was markedly impaired under both basal and insulin‐stimulated conditions. Maternal exercise did not restore basal glucose uptake, but normalised insulin‐stimulated glucose uptake in the epitrochlearis muscle of offspring from obese fathers (Fig. 4*B*).

**Figure 5:**
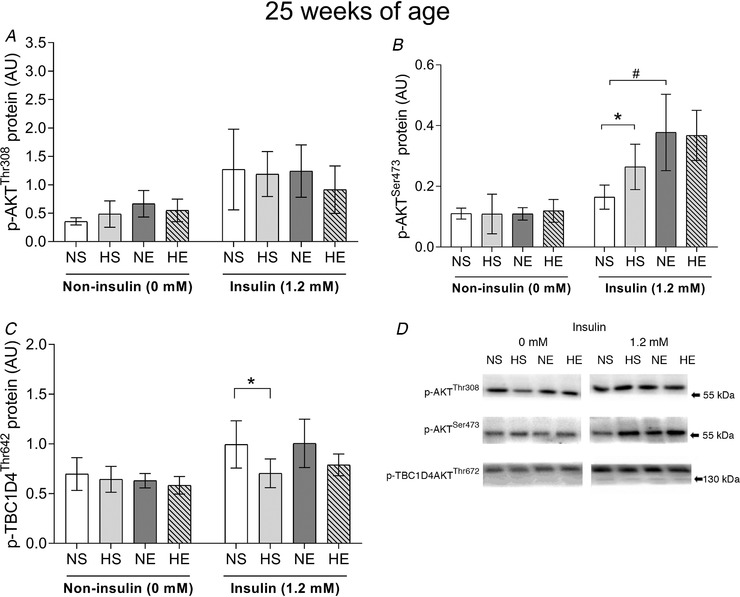
Insulin signalling in epitrochlearis muscle of adult offspring sired by control or obese fathers and sedentary or exercised mothers Phosphorylation of AKT Thr308 (*A*), Ser473 (*B*) and TBC1D4 Thr642 (*C*). Representative western blots show the quality and signal obtained with the respective antibodies; because they represent one animal, they do not necessarily represent an exact mean of their experimental group. NS, offspring sired by control diet fathers and sedentary mothers (*n* = 6); HS, offspring sired by obese fathers and sedentary mothers (*n* = 6); NE, offspring sired by control diet fathers and exercised mothers (*n* = 6); HE, offspring sired by obese fathers and exercised mothers (*n* = 6). Values are presented as mean ± SD. ^*^Paternal obesity effect, *P* < 0.05. # Maternal exercise effect, P < 0.05

Under insulin‐stimulated conditions, paternal obesity augmented p‐AKT^Ser473^ while p‐AKT^Thr308^ did not change (Fig. 5*A* and *B*). TBC1D4 phosphorylated protein (Thr642) was also lower in offspring of obese fathers (HS *vs*. NS) (Fig. 5*C*). Maternal exercise increased p‐AKT^Ser473^ under insulin‐stimulated conditions compared to the control group (Fig. 5*B*). However, maternal exercise was not able to overcome the lower phosphorylation of TBC1D4 (Thr642) in offspring sired by obese fathers (Fig. 5*C*). There were no changes in total AKT or TBC1D4 protein expression. In terms of glucose transporters, paternal obesity decreased GLUT1 expression but had no significant effect on GLUT4 (Fig. 4*C* and *D*). Maternal exercise alone increased GLUT1 and GLUT4 protein expressions (Fig. 4*C* and *D*), but did not have any effects when associated with paternal obesity.

### Mitochondrial respiration, flux control ratios, ROS production, protein expressions and CS activity

**Figure 6:**
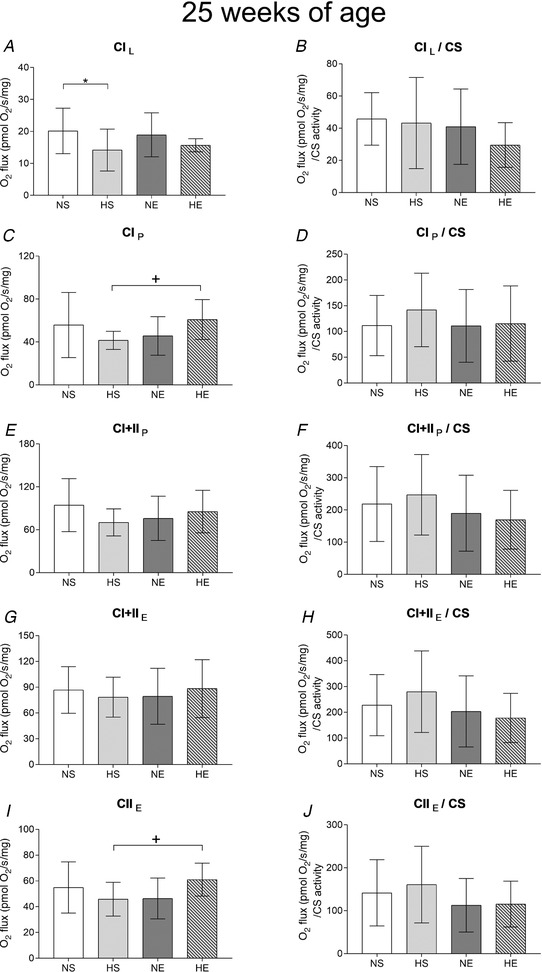
Mitochondrial respiration in plantaris muscle of adult offspring sired by control or obese fathers and sedentary or exercised mothers *A*, leak respiration through complex I (CI_L_). *C*, maximum oxidative phosphorylation (oxphos) capacity (*P*) through CI (CI_P_). *E*, *P* through complexes I+II (CI+II_P_). *G*, electron transport system (ETS) capacity (*E*) through CI+II (CI+II_E_). *I*, *E* through complex II (CII_E_). *B*, *D*, *F*, *H*, *J*: their respective measurements normalised by citrate synthase activity. NS, offspring sired by control diet fathers and sedentary mothers (*n* = 9); HS, offspring sired by obese fathers and sedentary mothers (*n* = 8); NE, offspring sired by control diet fathers and exercised mothers (*n* = 9); HE, offspring sired by obese fathers and exercised mothers (*n* = 10). Values are presented as mean ± SD. ^*^Paternal obesity effect, *P* < 0.05. ^+^Paternal obesity *vs*. Maternal exercise interaction, *P* < 0.05, followed by a least significant difference test (HS *vs*. HE).

**Figure 7:**
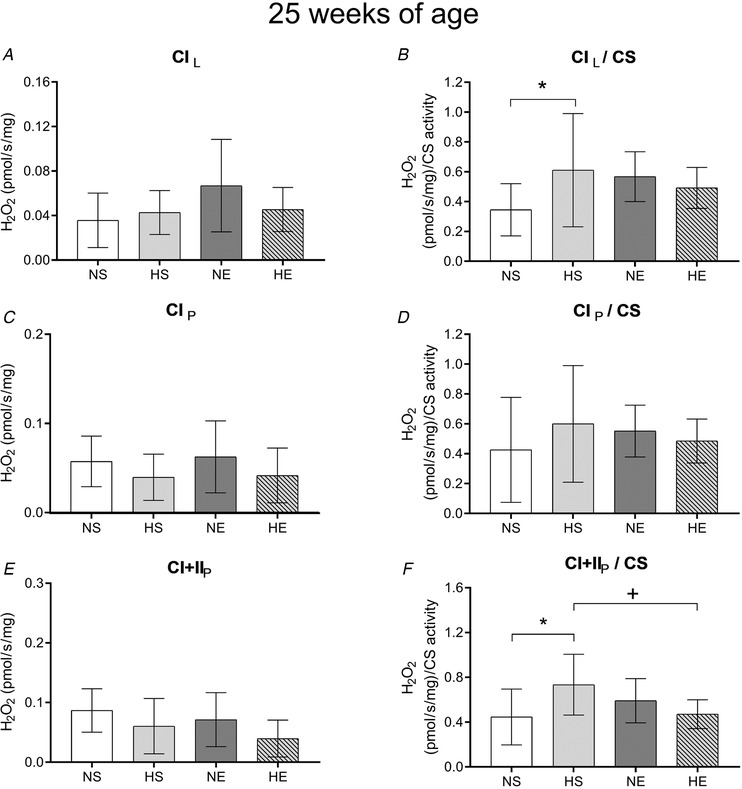
H_2_O_2_ production simultaneously measured during mitochondrial respiration in adult offspring sired by control or obese fathers and sedentary or exercised mothers *A*, hydrogen peroxide (H_2_O_2_) levels during leak respiration through complex I (CIL). *B*, H_2_O_2_ levels during maximum oxidative phosphorylation capacity through complex I (CIP). *C*, H_2_O_2_ levels during maximum oxidative phosphorylation capacity through complex I and II combined (CI+IIP). *B*, *D* and *F*: respective values normalised by citrate synthase (CS) activity. NS, offspring sired by control diet fathers and sedentary mothers (*n* = 9); HS, offspring sired by obese fathers and sedentary mothers (*n* = 8); NE, offspring sired by control diet fathers and exercised mothers (*n* = 9); HE, offspring sired by obese fathers and exercised mothers (*n* = 10). Values are presented as mean ± SD. ^*^Paternal obesity effect, *P* < 0.05. ^+^Paternal obesity *vs*. Maternal exercise interaction, *P* < 0.05, followed by a least significant difference test (HS *vs*. HE).

**Figure 8:**
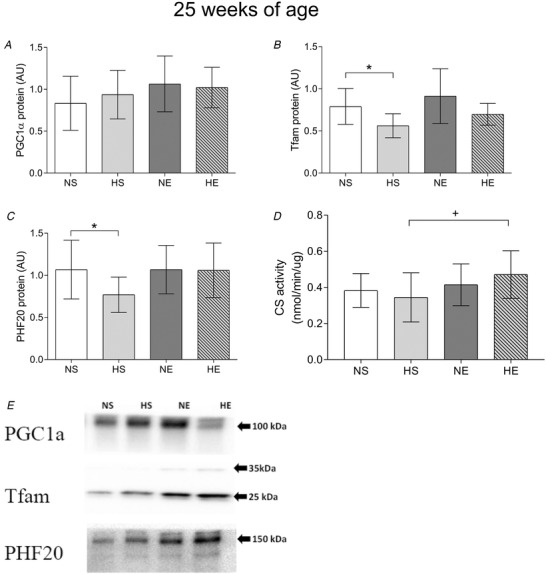
Protein expression and citrate synthase activity in plantaris muscle in adult offspring sired by control or obese fathers and sedentary or exercised mothers *A–C*, protein expression of PGC1α (*A*), Tfam (*B*) and PHF20 (*C*). Representative western blots show the quality and signal obtained with the respective antibodies; because they represent one animal, they do not necessarily represent an exact mean of their experimental group. *D*, citrate synthase activity in plantaris muscle. NS, offspring sired by control diet fathers and sedentary mothers (*n* = 10); HS, offspring sired by obese fathers and sedentary mothers (*n* = 10); NE, offspring sired by control diet fathers and exercised mothers (*n* = 10); HE, offspring sired by obese fathers and exercised mothers (*n* = 9). Values are presented as mean ± SD. ^*^Paternal obesity effect, *P* < 0.05. ^+^Paternal obesity *vs*. Maternal exercise interaction, *P* < 0.05, followed by a least significant difference test (HS *vs*. HE).

Paternal obesity did not change CS activity (Fig. 8) in adult offspring or most of the measured mitochondrial respiration components (Fig. 6*A*–*J*) except for leak through complex I, which was lower compared to the control group (Fig. 6*A*). Offspring born from obese fathers and exercised mothers had increased O_2_ flux in both CI_P_ and CII_E_ when compared to offspring sired by obese fathers and sedentary mothers (HE *vs*. HS; Fig. 6*C* and *I*, respectively). Maternal exercise increased CS activity in offspring sired by obese fathers (Fig. 7*D*). With regard to mitochondrial‐specific respiration, maternal exercise decreased CI+II_P_ and CII_E_ (Fig. 6*D*). Respiratory ratios did not change among the experimental groups (data not shown).

Mitochondrial H_2_O_2_ production was measured at the same time as mitochondrial respiration. Paternal obesity did not affect H_2_O_2_ emission (Fig. 7). Offspring sired by exercised mothers had lower H_2_O_2_ emission on the OXPHOS CI+II stage when compared to the HS group, while maternal exercise reduced H_2_O_2_ in CI+II_P_ when normalised by CS activity (Fig. 7). HS offspring had lower Tfam and PHF20 protein expression while PGC1α was not affected. Maternal exercise did not alter these protein expressions (Fig. 8*A*–*C*).

### Pancreas morphology

**Figure 9:**
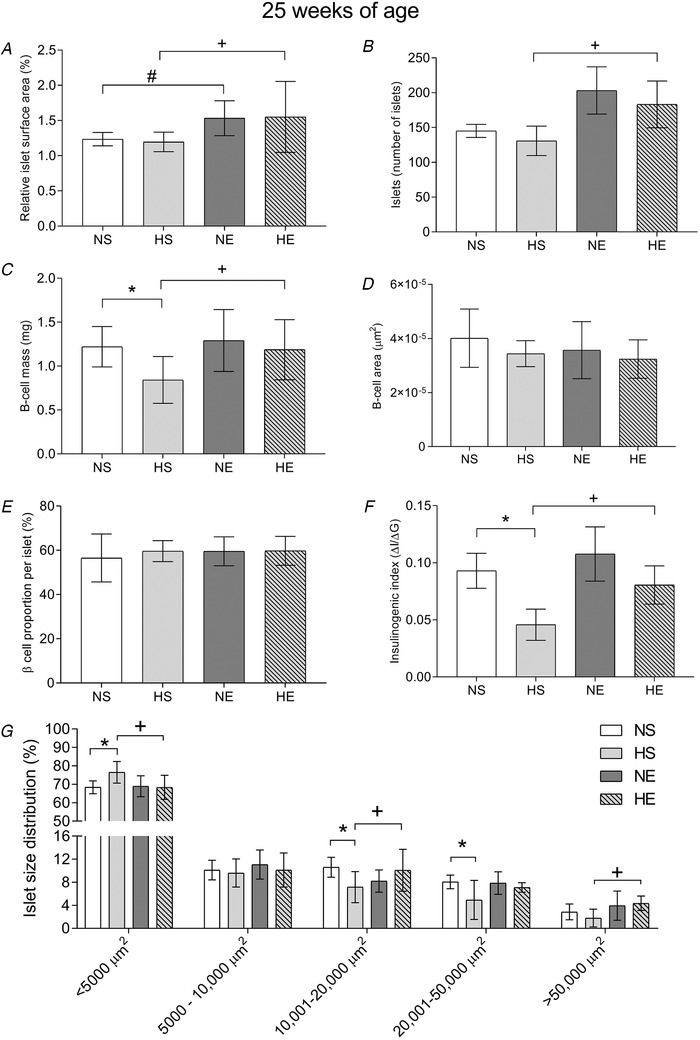
Pancreas morphology of adult offspring sired by control or obese fathers and sedentary or exercised mothers *A* and *B*, relative islet surface area expressed as a percentage of total pancreas surface area (*A*) and number of islets (*B*). *C*, beta cell mass was calculated as the product of whole pancreas weight before fixation and the ratio of insulin positive/total pancreas cross‐sectional area. Beta cell area (*D*) and beta cell proportion per islet (*E*). *F*, insulinogenic index, derived from IPGTT at 24 weeks of age with the following formula: insulinogenic index = AUC insulin (0–30 min)/AUC glucose (0–30 min) (Ng *et al*. 2010). *G*, islet distribution, arbitrarily classified according to their size: < 5000 µm^2^, 5000–10,000 µm^2^, 10,001–20,000 µm^2^, 20,001–50,000 µm^2^ and > 50,000 µm^2^. NS, offspring sired by control diet fathers and sedentary mothers (*n* = 10); HS, offspring sired by obese fathers and sedentary mothers (*n* = 10); NE, offspring sired by control diet fathers and exercised mothers (*n* = 10); HE, offspring sired by obese fathers and exercised mothers (*n* = 10). Values are presented as mean ± SD. ^*^Paternal obesity effect, *P* < 0.05. ^+^Paternal obesity *vs*. Maternal exercise interaction, *P* < 0.05, followed by a least significant difference test (HS *vs*. HE).

Relative islet surface area, number of islets, beta cell area and proportion were not affected by paternal obesity; however, beta cell mass and the insulinogenic index were lower (Fig. 9). The islet size distribution was also altered by paternal obesity, showing a higher number of small islets and a lower number of large and very large islets in offspring of obese fathers (Fig. 9*G*). Maternal exercise increased the relative islet surface area, the number of islets, and normalised the lower beta cell mass and insulinogenic index in adult offspring sired by obese fathers. Maternal exercise also normalised the increase in small islets (<5000 µm^2^) observed in adult offspring sired by obese fathers (Fig. 9*G*).

## Discussion

In this study, we have shown that exercise before and during pregnancy attenuated the lower insulin‐stimulated glucose uptake in offspring sired by obese fathers. Remarkably, maternal exercise also attenuated the lower insulin secretion *in vivo* in adult female offspring sired by obese fathers, which is probably explained by the higher beta cell mass.

Both maternal and paternal effects have the capacity to shape an offspring's phenotype (Uller, 2008). Like us, others have reported no effects of maternal physical activity (running wheel) on birth weight in rats (Carter *et al*. 2012, 2013; Stanford *et al*. 2015). However, exercise intensity and volume, as well as when the exercise starts, can influence neonatal weight (reviewed by Hopkins & Cutfield, 2011). Previously, we have reported no changes in birth weight with a similar exercise training protocol (Fidalgo *et al*. 2013). A lower intensity and volume of exercise towards the delivery period, as used in our study, has been reported not to alter birth weight (Hopkins & Cutfield, 2011). Maternal exercise normalised the reduced body mass in the adult offspring of obese fathers but had no impact on food intake, which was probably related to an increase lean tissue, as the soleus and plantaris muscles when compared to offspring sired by obese fathers. This phenotypical modulation suggests that further benefits could be associated with maternal exercise.

The positive effects of maternal exercise alone or associated with negative insults during pregnancy have previously been investigated in offspring. For instance, swimming exercise before and during pregnancy increased antioxidants and mitochondrial mass in the different regions of the offspring's brain (Marcelino *et al*. 2013). In terms of skeletal muscle, maternal physical activity (running wheel) increased mRNA expression of PGC1α in both male and female offspring 3 weeks postnatally (Raipuria *et al*. 2015). We and others have also reported positive metabolic outcomes in offspring sired by exercised dams that had experienced either maternal malnutrition or obesity. For example, maternal exercise (treadmill) before and during pregnancy attenuates the negative effects of perinatal undernutrition on maturation of the central nervous system (Falcao‐Tebas *et al*. 2012 *a*), as well as growth and development, and glucose homeostasis (Fidalgo *et al*. 2013; Falcao‐Tebas *et al*. 2012 *b*). Additionally, maternal physical activity (running wheel) before and during pregnancy completely prevented the low expression of PGC1α in adult offspring caused by maternal obesity (Laker *et al*. 2014). However, it is important to note that no studies have investigated the potential long‐term benefits of maternal exercise to attenuate the effects of paternal obesity in offspring.

We showed that paternal obesity compromises insulin sensitivity (estimated *in vivo* by IPITT) in adult offspring (Falcao‐Tebas *et al*. 2019). Surprisingly, we found that maternal exercise before and during gestation had no effects on *in vivo* insulin sensitivity in adulthood. In both mice and rats, maternal physical activity (running wheel) before and during gestation has been shown to improve insulin sensitivity in adult offspring compared to control (sedentary, normal chow fed parents) (Carter *et al*. 2012, 2013; Stanford *et al*. 2015). Changes in offspring phenotype may be dependent on the regime of exercise antenatally (Clapp *et al*. 2003, 2002). For instance, moderate to high volumes of exercise towards the end of pregnancy may lead to lower birth weight in humans (Hopkins & Cutfield, 2011). In rodents, free physical activity in a running wheel results in a higher exercise volume compared with the exercise usually performed on a treadmill. In fact, pregnant mice and rats can run up to 6–8 km per day in a running wheel (Carter *et al*. 2013; Stanford *et al*. 2015) compared to the ∼1 km achieved during an average session of 1 h of exercise training in our treadmill protocol. Importantly, it is possible to control exercise intensity with a treadmill, which is generally not addressed in studies using running wheel models. These differences in exercise protocol (volume and intensity) could explain, at least in part, the divergent findings for insulin sensitivity. Nevertheless, we showed that treadmill exercise before and during pregnancy can positively modulate the insulin sensitivity *ex vivo* of adult offspring.

Under insulin‐stimulated conditions, maternal exercise attenuated the effects of paternal obesity in adult offspring. Although we observed that GLUT4 protein expression was higher in offspring sired by exercised mothers, we did not assess GLUT4 translocation. We were unable to identify the insulin signalling responsible for the improvement in glucose uptake. In fact, both p‐AKT^Thr308^ and p‐TBC1D4^Thr642^ remained unchanged when compared with offspring born from obese fathers. Unexpectedly, paternal obesity increased p‐AKT^Ser473^ in adult offspring, with an even higher phosphorylation at this site in offspring sired by exercised mothers. Previous studies have reported an increased p‐Akt^Thr308^ at 6 weeks of age in offspring of obese fathers with no difference in p‐Akt^Ser473^ (Consitt *et al*. 2018). Note that only a small proportion of AKT needs to be phosphorylated (∼10%) to obtain maximum increases in AKT activity (Tan *et al*. 2012), so the relevance of increases in p‐AKT^Ser473^ above this level is not clear. Other phosphorylation sites might play a role in regulating insulin‐stimulated glucose uptake, such as TBC1D4 (Ser318, Ser341 and Ser704) and TBC1D1 (Thr596) (Treebak *et al*. 2014), as well as proximal insulin signalling (e.g. insulin receptor and insulin receptor substrates). Together, our results indicate that maternal exercise does not normalise *in vivo* insulin sensitivity (estimated by IPITT), but it does protect offspring in terms of insulin‐stimulated glucose uptake in isolated skeletal muscle.

There is evidence that skeletal muscle mitochondrial respiration can affect insulin sensitivity, although it is unclear whether mitochondrial dysfunction is a cause or consequence of insulin resistance in type 2 diabetes mellitus (Montgomery & Turner, 2015). We have previously demonstrated that maximal mitochondrial respiration was lower in the skeletal muscle of offspring sired by obese fathers (Falcao‐Tebas *et al*. 2019). In this study, offspring sired by obese fathers and exercised mothers increased the mitochondrial respiratory capacity (with saturating levels of ADP) as well as respiratory electron transfer system capacity, which suggests improved mitochondrial function (Gnaiger, 2007). Maternal exercise increased CS activity in offspring sired by obese fathers, which explains, at least in part, the observed decrease in CI+II_P_ and CII_E_ oxygen flux. It also suggests that changes in mitochondrial respiration were likely to be due to a higher mitochondrial content rather than mitochondrial capacity. The increased ROS production in mitochondrial‐specific respiration of adult offspring sired by obese fathers was normalised by exercised mothers (CI+II_P_/CS). This is important as an excess of mitochondrial ROS is considered to be a deleterious factor leading to insulin resistance in skeletal muscle (Houstis *et al*. 2006; Anderson *et al*. 2009).

There were no effects on protein expression of PGC1α, one of the main regulators of mitochondrial biogenesis. In young rats, maternal physical activity (running wheel) increased PGC1α gene expression compared with offspring born from control/sedentary mothers (Raipuria *et al*. 2015), but, like us, others have found that this effect does not seem to be sustained until adulthood (Laker *et al*. 2014). Tfam and PHF20 were lower in offspring sired by obese fathers and remained lower with maternal exercise. Tfam is an essential transcription factor of mitochondrial biogenesis (Campbell *et al*. 2012), while PHF20 regulates another important transcription factor, p53 (Cui *et al*. 2012), which plays a role in mitochondria. The lower expression of these proteins may suggest that the rate of transcription of genetic information might be compromised in adult offspring; however, this may not directly influence mitochondrial biogenesis. Therefore, in offspring sired by obese fathers, maternal exercise increases a marker of mitochondrial volume, without changes in markers of mitochondrial biogenesis and related transcription factors.

Maternal exercise attenuated the lower insulin secretion observed in adult female offspring sired by obese fathers. A possible link between improved insulin secretion and higher beta cell mass might explain, in part, such effects. We have previously shown in rats that treadmill exercise before and during pregnancy increases glucose‐stimulated insulin secretion in isolated islets from mothers (at the 3rd day of lactation) (Leandro *et al*. 2012), but whether similar effects would be observed in their offspring needs further investigation. One study showed that maternal exercise on a treadmill before and during pregnancy had no effects on insulin content in young (3 weeks of age) or adult (∼28 weeks of age) offspring (Quiclet *et al*. 2016). This study also demonstrated that maternal exercise improved insulin secretory capacity in young rats without any effects in adult rats. Of note, male offspring were used, and maternal exercise was kept at the same intensity until day 19 of pregnancy (Quiclet *et al*. 2016). This is concerning as human studies have shown that maintaining a high volume (60 min per session) of exercise until close to delivery can have negative impacts such as reduced birth weight (Hopkins & Cutfield, 2011). Similar deleterious findings were reported in animal studies that maintained a high volume or intensity of exercise during gestation, with effects on placental and fetal weight (Treadway & Young, 1989; Mottola *et al*. 1993).

With regard to pancreas morphology, we have previously shown that exercise training before and during gestation increased insulin secretion *in vitro* in the dams in late pregnancy (Leandro *et al*. 2012). Whether similar benefits would be observed in the offspring later in life was unknown. In the present study, maternal exercise normalised the percentage of islet size distribution, increased islet number and relative surface area in the offspring of obese fathers. It was not possible, however, to determine whether the effects of maternal exercise on islet number were due to cell proliferation or differentiation. This could be examined by staining proliferation markers (such as Ki‐67, a component of the mitotic chromosome) (Cuylen *et al*. 2016) and gene expression analysis. Nevertheless, this increase in islet number may also account for the attenuation of islet mass and insulin secretion in adult offspring born from exercised mothers and obese fathers.

It is important to acknowledge potential limitations of this research. First, we did not investigate whether the lower insulin‐stimulated glucose uptake was associated with a lower level of GLUT4 translocation to the membrane after insulin stimulation. Second, there are additional phosphorylation sites of AKT, TBC1D4 and TBC1D1 that may yield important information – such as insulin‐responsive phosphorylation sites in skeletal muscle of TBC1D1 at Thr596 and TBC1D4 at Ser318, Ser341 and Ser704 (Treebak *et al*. 2014). It is possible that we may have missed important potential regulation of insulin signalling at these sites. Third, experiments with isolated pancreatic islets would provide important understanding about the offspring's insulin secretory capacity. With isolated islets, it is possible to examine protein and gene expression relevant to beta‐cell function, e.g. glucose transporters and PDX1 (Chakrabarti *et al*. 2002). It is also possible to examine glucose‐ and arginine‐stimulated insulin secretion.

In summary, exercise before and during pregnancy had no effect on whole‐body insulin sensitivity in healthy offspring, but it normalised the insulin‐stimulated glucose uptake in isolated skeletal muscle in adult female offspring of obese fathers. The underlying mechanisms remain unclear. Maternal exercise increased a marker of mitochondrial content (i.e. CS activity) and normalised reactive oxygen species production from the skeletal muscle of female offspring sired by obese fathers, but there were no changes in markers of mitochondrial biogenesis and associated transcription factors. Remarkably, our protocol of treadmill exercise before and during pregnancy normalised the lower insulin secretion observed in female offspring of obese fathers, which might be due to an increase in the mass and number of beta cells and/or the area of pancreatic islets.

## Additional information

### Competing interests

The authors have no conflicts of interest.

### Author contributions

F.F.T. and G.K.M. conceived and designed the work; F.F.T., E.C.M. and J.K. conducted the acquisition of data; F.F.T., E.C.M., J.K., D.B. and G.K.M. performed analysis and interpretation of data for the work; F.F.T. drafted the manuscript; F.F.T., E.C.M., J.K. D.B. and G.K.M. revised and approved the final version.

### Funding

This study was supported by a research grant to Victoria University from the Australian Collaborative Research Network (CRN) and the Australian Institute for Musculoskeletal Science (AIMSS). Filippe Falcao‐Tebas was supported by an International Postgraduate Research Scholarship (Australian Government).

## Supporting information


**Statistical Summary Document**

## Data Availability

The data that support the findings of this study are available from the corresponding author upon reasonable request.
