# Supplementary material for: Maternal exercise attenuates the lower skeletal muscle glucose uptake and insulin secretion caused by paternal obesity in female adult rat offspring
Source: J Physiol. 2020 Jul 9;598(19):4251–70. doi: 10.1113/JP279582 (PMC7586952; doi:10.1113/JP279582)
Supplement: Supplementary file 1 — Statistical Summary Document [file TJP-598-4251-s001.pdf]

The Journal of Physiology

Statistical Summary Document

Manuscript Title: Maternal exercise attenuates the lower skeletal muscle glucose uptake and insulin secretion caused by paternal obesity in female adult rat offspring

Authors: Filippe Falcão-Tebas, Evelyn C Marin, Jujiao Kuang, David Bishop, Glenn K McConell

Animal model used, if applicable: Rat – Sprague Dawley

Underlying hypothesis: Maternal exercise attenuates the adverse metabolic effects of paternal obesity in rat offspring.

Definitions of 'n':
"n" refers to number of rats unless specified (e.g. n=litters).
Data presented as consecutive groups: NS, HS, NE, HE.

Statistical summary table:

Experimental question number*	Finding/ conclusion	Experimental location/ variable e.g. muscle, neocortex or genotype	Mean value (or other summary statistic)	SD (unless specified)	n val.	P**	Units	Data comparisons (ANOVA factors or specific groups)	Statistical test	Any other variable e.g. subjects' age or sex	Figure/ table in which data are presented	Comments e.g. observation
Any changes at PND1?	No changes	Pups born	Median:13.00,15.00, 12.00,13.50	Min-max: 10-16, 12-16, 11-15, 11-16	5 litters per group	0.3075	Number of pups	Dunn's multiple comparisons test: ns	Kruskal-Wallis		Table 2	Categorical data
	No changes	Female pups born	Median: 6.00, 8.00, 7.00, 7.50	Min-max: 4-9, 4-10, 6-9, 6-10	5 litters per group	0.1692	Number of pups	Dunn's multiple comparisons test: ns	Kruskal-Wallis		Table 2	Categorical data
	No changes	Male pups born	Median: 7.50, 8.00, 6.00, 6.00	Min-max: 5-10, 5-9, 3-9, 5-9	5 litters per group	0.3522	Number of pups	Dunn's multiple comparisons test: ns	Kruskal-Wallis		Table 2	Categorical data
	No changes	PND1 body weight females	7.23, 7.02, 8.09,6.93	0.2, 0.4, 1.2, 1.2	5 litters per group	0.1887, 0.2823, 0.0652	g	Effects: interaction, exercise, diet	ANOVA		Table 2	
	Diet effects	PND1 body weight males	7.69, 7.35, 8.55, 6.56	0.7, 0.4, 1.3, 1.0	5 litters per group	0.0527, 0.9293, 0.0100<br><br>p=0.0036	g	Effects: interaction, exercise, diet. NE vs HE	ANOVA		Table 2	
	No changes	PND1 blood glucose	4.67, 5.20, 5.25, 4.90	1.1, 0.6, 1.7, 1.3	5 litters per group	0.3939, 0.7817, 0.8584	mmol/l	Effects: interaction, exercise, diet	ANOVA		Table 2	
	No changes	Body weight	53.94, 52.56, 51.53, 47.36	1.7, 2.2, 2.1, 1.9	5 litters per group	0.4734, 0.0625, 0.1635	g	Effects: interaction, exercise, diet	ANOVA		Table 2	
	No effects	Body length	128.17, 128.04, 129.57, 125.35	2.95, 4.40, 4.19, 5.81	5 litters per group	0.3199, 0.7503, 0.2911	mm	Effects: interaction, exercise, diet	ANOVA		Table 2	

	No effects	Liver	2.29, 2.17, 2.25, 2.15	0.13, 0.17, 0.24, 0.40	5 litters per group	0.9316, 0.7971, 0.3521	g	Effects: interaction, exercise, diet	ANOVA		Table 2	
	No effects	Spleen	0.24, 0.22, 0.21, 0.20	0.02, 0.06, 0.04, 0.08	5 litters per group	0.8408, 0.3226, 0.5489	g	Effects: interaction, exercise, diet	ANOVA		Table 2	
	No effects	Heart	0.28, 0.25, 0.31, 0.25	0.04, 0.04, 0.07, 0.07	5 litters per group	0.5645, 0.5645, 0.0966	g	Effects: interaction, exercise, diet	ANOVA		Table 2	
	No effects	EDL	0.034, 0.021, 0.033, 0.031	0.0105, 0.006, 0.005, 0.0088	5 litters per group	0.0191; 0.8436; 0.0622	g	NS vs HS, NS vs NE, HS vs HE	ANOVA		Table 2	
	No effects	Soleus	0.03, 0.02, 0.03, 0.03	0.009, 0.007, 0.004, 0.007	5 litters per group	0.1756, 0.1756, 0.1756	g	Effects: interaction, exercise, diet	ANOVA		Table 2	
	No effects	Kidneys	0.31, 0.30, 0.30, 0.27	0.03, 0.04, 0.02, 0.04	5 litters per group	0.6437; 0.6437, 0.1765	g	Effects: interaction, exercise, diet	ANOVA		Table 2	
	HS lower, HE higher	Pancreas	0.21, 0.28, 0.22, 0.20	0.03, 0.04, 0.02, 0.04	5 litters per group	0.0083, 0.0033	g	NS vs HS, HS vs HE	ANOVA		Table 2	
	No effects	Blood glucose	8.87, 8.08, 9.03, 8.40	2.9, 4.1, 5.1, 4.6	5 litters per group	0.9670, 0.9012, 0.7139	mmol/L	Effects: interaction, exercise, diet	ANOVA		Table 2	
At 24	HS lower	Body mass	333.5, 299.1, 317.2, 315.3	44.3, 22.1, 38.4, 30.0	10,10,9,10	0.0329	g	NS vs HS	ANOVA		Table 3	
	No effects	Body length	24.3, 24.0, 24.6, 24.3	0.6, 0.3, 0.6, 0.3	10,10,9,10	>0.9999, 0.0545, 0.0545	cm	Effects: interaction, exercise, diet	ANOVA		Table 3	
	No effects	Abdominal circumference	18.3, 18.2, 17.9, 17.7	1.3, 0.6, 0.9, 0.9	10,10,9,10	0.8667, 0.1371, 0.6152	cm	Effects: interaction, exercise, diet	ANOVA		Table 3	
	HS lower, HE higher	Soleus	151.4, 130.9, 150.1, 149.6	18.7, 19.9, 10.5, 11.1	10,10,9,10	0.0063, 0.0119	mg	NS vs HS, HS v HE	ANOVA		Table 3	
	No effects	EDL	160.6, 149.2, 159.7, 153.4	22.1, 15.5, 16.5, 5.4	10,10,9,10	0.6229, 0.7501, 0.0940	mg	Effects: interaction, exercise, diet	ANOVA		Table 3	
	HS lower, HE higher	Plantaris	337.0, 314.4, 328.8, 335.0	36.0, 24.0, 38.7, 14.2	10,10,9,10	0.0465, 0.0370	mg	NS vs HS, HS vs HE	ANOVA		Table 3	
	NE lower	Tibialis anterior	669.4, 638.2, 566.6, 632.4	84.1, 34.5, 59.7, 48.4	10,10,9,10	0.0007	mg	NS vs NE	ANOVA		Table 3	
	No effects	Gastrocnemius	1.7, 1.6, 1.7, 1.6	0.230, 0.123, 0.149, 0.06	10,10,9,10	0.9999, 0.9999, 0.0495	g	Effects: interaction, exercise, diet	ANOVA		Table 3	
	HE higher	Liver	8.5, 8.1, 8.7, 9.1	0.9, 0.6, 1.2, 1.3	10,10,9,10	0.0371	g	HS vs HE	ANOVA		Table 3	
	Diet effect	Pancreas	1.4, 1.2, 1.4, 1.2	0.3, 0.3,0.3, 0.02	10,10,9,10	0.9999, 0.9999, 0.0213	g	Effects: interaction, exercise, diet	ANOVA		Table 3	

	No effects	Retro fat	6.2, 6.9, 6.4, 6.4	0.9, 1.9, 4.2, 1.5	10,10,9,10	0.6516, 0.8464, 0.6516	g	Effects: interaction, exercise, diet	ANOVA		Table 3	
	HS lower, HE higher	Kidney	0.938, 0.874, 0.92, 0.949	0.065, 0.078, 0.063, 0.073	10,10,9,10	0.0491, 0.0224	g	NS vs HS, HS v HE	ANOVA		Table 3	
	No effects	Heart	0.986, 0.928, 0.976, 1.006	0.031, 0.066, 0.096, 0.077	10,10,9,10	0.0603, 0.1426, 0.5408	g	Effects: interaction, exercise, diet	ANOVA		Table 3	
	No effects	Fasting glucose	5.66, 5.63, 5.90, 5.75	0.3, 0.9, 1.1, 0.4	10,10,9,10	0.8021, 0.4537, 0.7071	mmol/L	Effects: interaction, exercise, diet	ANOVA		Table 3	
	No effects	Fasting insulin	0.15, 0.13, 0.14, 0.11	0.04, 0.1, 0.1, 0.1	10,10,9,10	0.8612, 0.6005, 0.3845	ng/mL	Effects: interaction, exercise, diet	ANOVA		Table 3	
11-12 weeks	No effects	Glucose AAC	151.6, 133.6, 128.6, 136.2	18.7, 34.6, 32.7, 12.4	10,10,9,10	0.3587, 0.2949, 0.7862	AU	Effects: interaction, exercise, diet	ANOVA		Figure 2	
	No effects	Glucose AUC (0-30)	330.0, 357.3, 327.4, 327.8	30.0, 34.9, 44.6, 53.6	10,10,9,10	0.7392, 0.1178, 0.5863	AU	Effects: interaction, exercise, diet	ANOVA		Figure 2	
	HS higher	Glucose AUC (30-90)	478.5, 533.4, 460.1, 507.4	22.6, 56.7, 46.2, 53.1	10,10,9,10	0.0216	AU	NS vs HS	ANOVA		Figure 2	
	HS higher	Glucose AUC (Total)	823.0, 893.6, 787.5, 835.1	55.5, 37.1, 89.6, 81.4	10,10,9,10	0.0477	AU	NS vs HS	ANOVA		Figure 2	
	HE lower	Insulin AUC (0-30)	27.1, 32.7, 32.6, 16.9	12.6, 20.4, 10.6, 9.7	10,10,9,10	0.0167	AU	HS vs HE	ANOVA		Figure 2	
	No effects	Insulin AUC (30-90)	9.9, 11.5, 12.7, 10.5	3.4, 1.8, 6.2, 2.7	10,10,9,10	0.1932, 0.8081, 0.9299	AU	Effects: interaction, exercise, diet	ANOVA		Figure 2	
	HE lower	Insulin AUC (total)	37.0, 44.3, 45.4, 27.4	14.4, 20.9, 14.7, 12.4	10,10,9,10	0.0222	AU	HS vs HE	ANOVA		Figure 2	
23-24 weeks	HS lower	Glucose AAC	97.4, 75.9, 95.2, 92.7	20.5, 11.9, 27.9, 12.3	10,10,9,10	0.0081	AU	HS vs HE	ANOVA		Figure 3	
	No effects	Glucose AUC (0-30)	333.9, 346.9, 334.5, 344.4	40.7, 28.6, 20.0, 35.9	10,10,9,10	0.6593, 0.8531, 0.5084	AU	Effects: interaction, exercise, diet	ANOVA		Figure 3	
	HS higher	Glucose AUC (30-90)	478.8, 532.5, 486.0, 502.5	52.0, 32.6, 28.4, 46.5	10,10,9,10	0.0464	AU	NS vs HS	ANOVA		Figure 3	
	HS higher	Glucose AUC (Total)	812.6, 879.4, 820.5, 846.9	58.9, 37.1, 41.5, 80.3	10,10,9,10	0.0218	AU	NS vs HS	ANOVA		Figure 3	
	No effects	Insulin AUC (0-30)	35.8, 19.9, 34.7, 24.0	16.7, 10.3, 16.2, 13.6	10,10,9,10	0.7152, 0.5242, 0.0494	AU	Effects: interaction, exercise, diet	ANOVA		Figure 3	
	No effects	Insulin AUC (30-90)	20.1, 13.2, 19.4, 20.2	19.6, 4.4, 14.7, 4.9	10,10,9,10	0.4698, 0.4819, 0.5584	AU	Effects: interaction, exercise, diet	ANOVA		Figure 3	
	HE lower	Insulin AUC (total)	55.9, 33.1, 54.1, 44.2	28.3, 8.4, 24.2, 11.7	10,10,9,10	0.0383, 0.0302	AU	NS vs HS, HS vs HE	ANOVA		Figure 3	

The Journal of Physiology Statistical Summary Document

	No effects	Non-insulin (Soleus)	2.69, 2.09, 2.33, 2.71	0.33, 0.62, 0.89, 0.83	10,10,9,10	0.1251, 0.6737, 0.7182	µmol/g/h	Effects: interaction, exercise, diet	ANOVA		Figure 4	
	No effects	Insulin (Soleus)	3.60, 4.04, 3.62, 3.45	0.47, 0.93, 1.28, 0.66	10,10,9,10	0.4335, 0.4694, 0.7156	µmol/g/h	Effects: interaction, exercise, diet	ANOVA		Figure 4	
	HS lower	Non-insulin (EPI)	1.73, 0.96, 1.46, 1.37	0.45, 0.32, 0.29, 0.25	10,10,9,10	0.0010	µmol/g/h	NS vs HS	ANOVA		Figure 4	
	HS lower, NE higher	Insulin (EPI)	2.39, 1.54, 2.51, 2.34	0.44, 0.44, 0.40, 0.72	10,10,9,10	0.0099, 0.0182	µmol/g/h	NS vs HS, HS vs HE	ANOVA		Figure 4	
	HS lower, NE higher	GLUT1	0.35, 0.20, 0.50, 0.31	0.15, 0.11, 0.25, 0.14	6, 6, 6, 6	0.0488, 0.0395	AU	NS vs HS, NS vs NE	ANOVA		Figure 4	
	NE higher	GLUT4	1.0, 0.9, 1.4, 1.2	0.3, 0.3 0.3, 0.2	6, 6, 6, 6	0.0403, 0.0548	AU	NS vs NE, HS vs HE	ANOVA		Figure 4	
	NE higher	Non-insulin (p-AKT 308)	0.36, 0.48, 0.67, 0.55	0.06, 0.23, 0.23, 0.20	6, 6, 6, 6	0.0120	AU	NS vs NE	ANOVA		Figure 5	
	No effects	Insulin (p-AKT 308)	1.27, 1.19, 1.24, 0.91	0.71, 0.40, 0.46, 0.42	6, 6, 6, 6	0.5601, 0.4757, 0.3423	AU	Effects: interaction, exercise, diet	ANOVA		Figure 5	
	No effects	Non-insulin (p-AKT 473)	0.11, 0.11, 0.11, 0.12	0.02, 0.07, 0.02, 0.04	6, 6, 6, 6	0.7293, 0.7778, 0.7921	AU	Effects: interaction, exercise, diet	ANOVA		Figure 5	
	HS higher, NE higher	Insulin (p-AKT 473)	0.16, 0.26, 0.38, 0.37	0.04, 0.07, 0.13, 0.08	6, 6, 6, 6	0.0498, 0.0003	AU	NS vs HS, NS vs NE	ANOVA		Figure 5	
	No effects	Non-insulin (p-TBC1D4 642)	0.70, 0.64, 0.63, 0.58	0.16, 0.13, 0.07, 0.09	6, 6, 6, 6	0.9478, 0.2101, 0.3204	AU	Effects: interaction, exercise, diet	ANOVA		Figure 5	
	HS lower	Insulin (p-TBC1D4 642)	0.99, 0.70, 1.01, 0.79	0.24, 0.14, 0.24, 0.11	6, 6, 6, 6	0.0169	AU	NS vs HS	ANOVA		Figure 5	
	HS lower	CI L	20.14, 14.18, 19.02, 15.71	7.10, 6.55, 7.31, 2.58	10,10,9,10	0.0372	pmol O2/s/mg	NS vs HS	ANOVA		Figure 6	
	No effects	CI L / CS	45.78, 43.24, 40.94, 29.54	16.30, 28.31, 23.40, 13.87	10,10,9,10	0.5586, 0.2259, 0.3595	pmol O2/ s/ mg /CS	Effects: interaction, exercise, diet	ANOVA		Figure 6	
	HE higher	CI P	55.82, 41.50, 45.62, 59.93	30.37, 8.44, 17.96, 20.10	10,10,9,10	0.0453	pmol O2/s/mg	NS vs HS	ANOVA		Figure 6	
	No effects	CI P / CS	111.62, 141.96, 110.87, 108.43	58.52, 71.37, 70.61, 70.50	9, 8, 9, 8	0.5933, 0.5720, 0.4710	pmol O2/ s/ mg /CS	Effects: interaction, exercise, diet	ANOVA		Figure 6	
	No effects	CI + II P	94.44, 70.24, 76.02, 85.44	37.00, 18.93, 30.91, 29.72	9, 9, 9, 8	0.1064, 0.8741, 0.4701	pmol O2/s/mg	Effects: interaction, exercise, diet	ANOVA		Figure 6	
	No effects	CI + II P / CS	218.53, 247.19, 189.99, 169.56	116.25, 125.03, 117.79, 91.19	9, 8, 9, 7	0.5441, 0.1946, 0.9188	pmol O2/ s/ mg /CS	Effects: interaction, exercise, diet	ANOVA		Figure 6	
	No effects	CI + II E	86.80, 78.46, 79.48, 88.38	27.11, 23.24, 32.53, 33.74	9, 9, 9, 8	0.3916, 0.8967, 0.9780	pmol O2/s/mg	Effects: interaction, exercise, diet	ANOVA		Figure 6	

	No effects	CI + II E / CS	228.03, 280.26, 203.46, 178.24	118.62, 158.34, 137.83, 95.47	9, 8, 9, 7	0.4040, 0.1768, 0.7697	pmol O2/ s/ mg /CS	Effects: interaction, exercise, diet	ANOVA		Figure 6	
	HE higher	CII E	54.91, 45.83, 46.41, 61.06	19.84, 13.14, 15.89, 12.74	9, 9, 9, 9	0.0474	pmol O2/s/mg	HS vs HE	ANOVA		Figure 6	
	No effects	CII E / CS	141.67, 160.86, 112.73, 115.47	77.12, 89.36, 62.30, 53.47	9, 8, 9, 7	0.7475, 0.1529, 0.6680	pmol O2/ s/ mg /CS	Effects: interaction, exercise, diet	ANOVA		Figure 6	
	No effects	H2O2 - CI L	0.04, 0.04, 0.07, 0.05	0.02, 0.02, 0.04, 0.02	6, 8, 10, 7	0.1988, 0.1283, 0.5093	pmol O2/s/mg	Effects: interaction, exercise, diet	ANOVA		Figure 7	
	No effects	H2O2 - CI P	0.06, 0.04, 0.06, 0.04	0.03, 0.03, 0.04, 0.03	6, 8, 10, 9	0.8983, 0.7583, 0.1054	pmol O2/s/mg	Effects: interaction, exercise, diet	ANOVA		Figure 7	
	No effects	H2O2 - CI + II P	0.09, 0.06, 0.07, 0.04	0.04, 0.05, 0.05, 0.03	6, 8, 10, 7	0.8603, 0.2412, 0.0643	pmol O2/s/mg	Effects: interaction, exercise, diet	ANOVA		Figure 7	
	HS higher	H2O2 / CS - CI L	0.35, 0.61, 0.57, 0.49	0.17, 0.38, 0.17, 0.14	6, 8, 10, 7	0.0479	pmol O2/s/mg	NS vs HS	ANOVA		Figure 7	
	No effects	H2O2 / CS - CI P	0.43, 0.60, 0.55, 0.49	0.35, 0.39, 0.17, 0.15	6, 7, 10, 6	0.2621, 0.9580, 0.6149	pmol O2/s/mg	Effects: interaction, exercise, diet	ANOVA		Figure 7	
	HS higher, HE lower	H2O2 /CS - CI + II P	0.45, 0.73, 0.59, 0.47	0.25, 0.27, 0.20, 0.13	6, 7, 10, 6	0.0253, 0.0394	pmol O2/s/mg	NS vs HS, HS vs HE	ANOVA		Figure 7	
	No effects	PHF20	1.07, 0.77, 1.07, 1.06	0.35, 0.21, 0.29, 0.32	9, 8, 8, 7	0.2561, 0.2581, 0.2287	AU	NS vs HS	ANOVA		Figure 8	
	HS lower	PGC1-alfa	0.83, 0.94, 1.06, 1.02	0.32, 0.29, 0.33, 0.24	8, 8, 8, 7	0.5065, 0.1549, 0.7803	AU	Effects: interaction, exercise, diet	ANOVA		Figure 8	
	HS lower	Tfam	0.79, 0.56, 0.91, 0.70	0.21, 0.14, 0.32, 0.13	7, 8, 7, 7	0.0493	AU	NS vs HS	ANOVA		Figure 8	
	HE higher	CS activity	0.38, 0.35, 0.41, 0.47	0.09, 0.14, 0.12, 0.13	10, 9, 10, 7	0.0424	AU	HS vs HE	ANOVA		Figure 8	
	NE higher, HE higher	Relative islet surface area	1.24, 1.20, 1.53, 1.55	0.09, 0.14, 0.25, 0.50	10, 8, 8, 8	0.0353, 0.0179	%	NS vs NE, HS vs HE	ANOVA		Figure 9	
	NE higher, HE higher	Islet number	145.11, 130.78, 197.00, 183.25	9.44, 21.17, 31.36, 33.62	9, 9, 7, 8	0.0003, 0.0002	n of islets	NS vs NE, HS vs HE	ANOVA		Figure 9	
	HS lower, HE higher	b-cell mass	1.22, 0.84, 1.29, 1.19	0.23, 0.27, 0.35, 0.34	9, 8, 8, 8	0.0146, 0.0292	mg	NS vs HS, HS vs HE	ANOVA		Figure 9	
	No effects	b-cell area (data presented as $x10^{-5}$)	4.0, 3.4, 3.6, 3.2	1.1, 0.5, 1.1, 0.7	10, 8, 8, 8	0.6945, 0.3000, 0.1475	µm2	Effects: interaction, exercise, diet	ANOVA		Figure 9	
	No effects	b-cell proportion per islet (%)	56.53, 59.65, 52.94, 53.10	10.84, 4.73, 20.77, 20.83	9, 8, 9, 9	0.5861, 0.5634, 0.5400	%	Effects: interaction, exercise, diet	ANOVA		Figure 9	
	HS lower, HE higher	Insulinogenic index	0.09, 0.05, 0.11, 0.08	0.02, 0.01, 0.02, 0.02	6, 6, 6, 6	0.0002, 0.0030	ΔI/ΔG	NS vs HS, HS vs HE	ANOVA		Figure 9	

	HS higher, HE lower	Islet size distribution <5000µm2	68.40, 76.54, 68.96, 68.36	3.48, 5.82, 5.66, 6.54	10, 9, 8, 7	0.0025, 0.0050	%	NS vs HS, HS vs HE	ANOVA		Figure 9	
	No effects	Islet size distribution 5000-10000µm2	10.10, 9.60, 11.07, 10.12	1.69, 2.43, 2.52, 2.95	10, 9, 8, 7	0.7891, 0.3736, 0.3820	%	Effects: interaction, exercise, diet	ANOVA		Figure 9	
	HS higher, HE lower	Islet size distribution 10000-20000µm2	10.58, 7.16, 8.21, 10.08	1.73, 2.69, 1.93, 3.63	10, 9, 8, 7	0.0059, 0.0282	%	NS vs HS, HS vs HE	ANOVA		Figure 9	
	HS lower	Islet size distribution 20000-50000µm2	8.06, 4.93, 7.86, 7.09	1.18, 3.38, 1.95, 0.82	10, 9, 8, 7	0.0031	%	NS vs HS	ANOVA		Figure 9	
	HE higher	Islet size distribution >50000µm2	2.85, 1.78, 3.94, 4.35	1.36, 1.55, 2.53, 1.26	10, 9, 8, 7	0.0063	%	HS vs HE	ANOVA		Figure 9	

*You may use multiple lines for the same question to indicate multiple comparisons

** Authors may wish to make the text bold where p is considered significant against a stated confidence limit.
